# Factors Influencing Decision Making for Carotid Endarterectomy versus Stenting in the Very Elderly

**DOI:** 10.3389/fneur.2017.00220

**Published:** 2017-05-26

**Authors:** Sung Hyuk Heo, Cheryl D. Bushnell

**Affiliations:** ^1^Department of Neurology, School of Medicine, Kyung Hee University, Seoul, South Korea; ^2^Department of Neurology, Wake Forest School of Medicine, Winston-Salem, NC, United States

**Keywords:** carotid stenosis, carotid endarterectomy, carotid stenting, stroke, elderly

## Abstract

As the population ages worldwide, the number of elderly patients with carotid stenosis is also increasing. There have been many large clinical trials comparing carotid endarterectomy (CAE) versus stenting, but the inclusion criteria (i.e., symptomatic or asymptomatic), stenting methods (i.e., protection device), and primary end point (i.e., the definition of myocardial infarction and follow-up period) were different between trials. Therefore, the interpretation of those results is difficult and requires attention. When it comes to age, the patients older than 80 years were excluded or stratified to a high risk group in previous landmark trials. However, a recent guideline recommended that endarterectomy may be associated with lower stroke risk compared with carotid artery stenting in patients older than 70 years with symptomatic carotid disease. The annual risk of stroke in individuals with asymptomatic carotid stenosis is about 1–3% but the risk is about 4–12% with symptomatic stenosis without carotid intervention. Although the outcome of CAE is better than that of carotid stenting in patients older than 70 years, the perioperative risk is higher in older patients. Therefore, it is important to classify high risk patients and consider underlying disability and life expectancy of very elderly patients before deciding whether to undergo a carotid intervention. In addition, we should also consider that the stroke rate with intensive medical treatment is unknown and is currently being investigated in randomized controlled trials. Intensive medical treatment includes high intensity statins, diabetes and blood pressure control, and aggressive antiplatelet treatment. The aim of this review is to report the factors that may be responsible for the variability in the treatment of carotid stenosis, particularly in the elderly population. This will allow the readers to integrate the current available evidence to individualize the treatment of carotid stenosis in this challenging population.

## Introduction

Atherosclerosis is a chronic inflammatory disease of the arterial wall that slowly progresses pathologically causing arterial stenosis, resulting in cerebrovascular or coronary artery diseases (1, 2). It usually arises at the bifurcation of blood vessels having a disruption of laminar flow, and the carotid bulb or sinus is the region where most atherosclerotic plaques are found. Stroke caused by carotid stenosis is more severe in neurological deficits and has a high risk of recurrence (3).

The basic treatment of carotid atherosclerosis is management of risk factors such as hypertension, dyslipidemia, diabetes, and smoking with lifestyle modification, and antiplatelet medications. However, current guidelines recommend carotid revascularization procedures such as carotid endarterectomy (CEA) or carotid artery stenting (CAS) depending on the presence of symptoms and the degree of stenosis (4, 5). Various other conditions such as age and patient factors, features of atherosclerotic lesions, anatomical characteristics of cerebral and extracerebral vessels, and medical comorbidity should also be considered. In the past, either because of the concern of excessive risk or decreased post-procedure life expectancy, elderly patients (usually >80 years) have been excluded from randomized trials (6–10). However, recent trials showed more favorable outcomes after CEA versus CAS in elderly patients, so the guideline changed the recommendation for symptomatic older patients (i.e., older than ≈70 years) to undergo CEA rather than CAS (4, 11–13).

In western countries, there has been a remarkable increase in the population of those over 80 years. In non-western countries, one study predicts there is more than a 50% probability that by 2030, national female life expectancy will break the 90 year barrier in South Korea, a level that was deemed unattainable by some at the turn of the twenty-first century (14). Stroke is the fifth leading cause of death in the United States and is a major cause of serious disability for the elderly (15). Extracranial carotid artery disease is responsible for up to 20% of these strokes and accounts for a higher proportion in elderly patients (16, 17). The numbers of elderly patients with carotid stenosis will increase exponentially, and the results of medical treatment and CAS are getting better. Therefore, the selection of treatment method for carotid stenosis in elderly patients will be more complex.

In this review article, our aim is to describe the selection criteria for the appropriate treatment methods in elderly patients and the considering factors that affect it, based on current literature.

## Short-Term and Long-Term Outcomes after Carotid Revascularization in Symptomatic and Asymptomatic Patients

### Treatment of Asymptomatic Carotid Stenosis

Asymptomatic carotid artery stenosis is a very important health issue, as out of the 135,701 carotid revascularizations performed in the US in 2005, 122,986 (92%) were for asymptomatic carotid artery stenosis (18). Since the results of large clinical trials comparing CEA with drug therapy in the treatment of asymptomatic carotid stenosis was published in 1990s, CAS, optimal medical treatment, and variations of surgical approaches to CEA have been also developed (8, 19). In the Asymptomatic Carotid Atherosclerosis Stenosis (ACAS) and Asymptomatic Carotid Surgery (ACST) trials, the 5-year risk of stroke or procedural morbidity was estimated to be 6.5% for CEA patients and 11.0% for patients treated medically among asymptomatic patients with carotid artery stenosis of greater than 60% (8, 20). The meta-analysis of the Veterans Administration Cooperative Study (VA), ACAS, and ACST trials shows that in patients with asymptomatic carotid stenosis, despite about a 3% perioperative stroke or death rate, CEA reduces the risk of ipsilateral stroke and any stroke, by approximately 30% over 3 years (21). In the case of asymptomatic stenosis, CEA is beneficial if the incidence of surgical complication is less than 3% in patients with stenosis of 60% or more, but some researchers claim that the complication rate should be lower than 3% because of improved drug treatment results. In recent studies, the annual rate of stroke in medically treated patients with an asymptomatic carotid artery stenosis has fallen to ≤1% (22, 23).

The National Institute of Neurological disorders and Stroke-sponsored Carotid Revascularization Endarterectomy versus Stenting Trial (CREST) included symptomatic patients only at baseline, but expanded inclusion criteria for asymptomatic patients (final proportion of asymptomatic patients was 47.2% of the total) (12). In the analysis among patients with asymptomatic carotid stenosis, there was no difference in the primary outcome between CAS and CEA [5.6 versus 4.9%; hazard ratio (HR) 1.17, 95% confidence interval (CI) 0.69–1.98, *p* = 0.56]. All stroke cases within 30 days and the occurrence of ipsilateral stroke between 30 days and 4 years were 4.5% in CAS group and 2.7% in CEA group so there was no statistical difference (HR 1.86; 95% CI 0.95–3.66, *p* = 0.07). The incidence of ipsilateral stroke between 30 days and 4 years (i.e., durability of the intervention) was similar, with 2.0% in the CAS group and 2.4% in the CEA group. The durability was favorable in long-term follow-up for both groups (12). The Asymptomatic Carotid Trial (ACT)-1, which compared the outcomes of CEA versus CAS in patients with asymptomatic severe carotid artery stenosis who were at standard risk for surgical complications, revealed that CAS was non-inferior to CEA with regard to the primary composite end point (3.8 and 3.4%, respectively; *p* = 0.01 for non-inferiority), but this study excluded patients who were 80 years of age or older (24).

ACST-1 was a 10-year follow-up study that investigated the long-term prognosis after the ACST trial (25). The incidence of stroke or death within 30 days of CEA was 3.0% (95% CI 2.4–3.9) in all patients undergoing CEA. Excluding perioperative events and non-stroke mortality, stroke risks (immediate versus deferred CEA) were 4.1 versus 10.0% at 5 years (gain 5.9%, 95% CI 4.0–7.8) and 10.8 versus 16.9% at 10 years (gain 6.1%, 2.7–9.4); ratio of stroke incidence rates 0.54, 95% CI 0.43–0.68, *p* < 0.0001. Combining perioperative events and strokes, net risks were 6.9 versus 10.9% at 5 years (gain 4.1%, 2.0–6.2) and 13.4 versus 17.9% at 10 years (gain 4.6%, 1.2–7.9). In spite of stroke or death associated with surgery, the surgical outcome was excellent. In addition, more than half of the patients with stroke (166/287) died of or were disabled by stroke, and the proportional reduction in disabling or fatal stroke seemed to be similar to that for any stroke.

### Treatment for Symptomatic Carotid Stenosis

In the North American Symptomatic Carotid Endarterectomy Trial (NASCET), CEA reduced the two-year risk of ipsilateral stroke from 26% in the medical arm to 9% in the surgical group, yielding an absolute risk reduction of 17% (6). This result was similar to other randomized clinical trials comparing CEA with medical treatment such as the European Carotid Surgery Trial (ECST) and the Veterans Affairs Cooperative Study (VACS) (7, 26). Pooled analysis of the VACS, NASCET, and ECST found a 30-day stroke and death rate of 7.1% in the CEA arm in patients with transient ischemic attack (TIA) or stroke within 6 months, and CEA was highly beneficial in those with 70–99% stenosis (27). The role of CEA is less clear in the symptomatic patients with moderate stenosis (50–69%). In NASCET patients with a stenosis of 50–69%, the 5-year rate of any ipsilateral stroke was 15.7% in CEA arm compared with 22.2% in medical arm (*p* = 0.045).

Comparative studies between CEA and CAS such as the Stent-Protected Angioplasty of the Carotid Artery versus Endarterectomy (SPACE), the Endarterectomy Versus Angioplasty in patients with Symptomatic Severe carotid Stenosis trial (EVA-3S), and the International Carotid Stenting Study (ICSS) showed better periprocedural outcomes in CEA rather than CAS, especially in older patients (11, 13, 28, 29). Meta-analysis from these trials revealed any stroke or death occurred significantly more often in the CAS group (8.9%) than in the CEA group (5.8%) in the first 120 days after randomization [risk ratio 1.53 (95% CI 1.20–1.95), *p* = 0.0006] (11). Subgroup analysis from ICSS trial using MRI showed the presence of at least one new ischemic brain lesion on diffusion weighted imaging 1–3 days after treatment was more common in CAS group than CEA group (50 versus 17%; *p* < 0.0001) (30).

However, because CAS was potentially favorable in the Carotid And Vertebral Artery Transluminal Angioplasty Study (CAVATAS) and the Stenting and Angioplasty with Protection in Patients at High Risk of Endarterectomy (SAPPHIRE) trials, CREST was designed to compare the efficacy of CAS with that of CEA (31, 32). In this non-inferiority trial, 2,502 symptomatic and asymptomatic patients with carotid stenosis were enrolled and randomized to CAS or CEA. There was no significant difference in the composite primary outcome [30-day rate of stroke, death, and myocardial infarction (MI) and 4-year ipsilateral stroke] in patients treated with CAS versus CEA (7.2 versus 6.8%; HR for stenting, 1.1; 95% CI 0.81–1.51; *p* = 0.51). In symptomatic patients, the 4-year rate of the primary end point was 8.6% with CAS versus 8.4% with CEA (HR, 1.08; 95% CI 0.74–1.59; *p* = 0.69) (12, 33). Periprocedural rates of individual components of the end points differed between the CAS group and CEA group: for death (0.7 versus 0.3%, *p* = 0.18), for stroke (4.1 versus 2.3%, *p* = 0.01), for MI (1.1 versus 2.3%, *p* = 0.03), and for cranial nerve palsies (0.3 versus 4.7%; *p* = 0.0001).

In most studies evaluating long-term outcome after CEA versus CAS, there was no difference in major vascular outcomes between the treatment methods after the periprocedural period (34–39). In the SPACE trial, the rate of ipsilateral ischemic strokes up to 2 years including any periprocedural strokes or deaths after procedure was similar for CEA and CAS groups (9.5 and 8.8%; HR 1.10 (0.75–1.61); log rank *p* = 0.62). In addition, the rate of ipsilateral ischemic stroke within 31 days and 2 years was not different between CAS (2.2%) and CEA (1.9%) (35). In the 4-year follow-up of EVA-3S trial, the risk of ipsilateral stroke was low and similar in both treatment groups after the periprocedural period (37). In the ICSS trial, the cumulative 5-year risk of fatal or disabling stroke did not differ significantly between CAS and CEA groups (6.4 versus 6.5%; HR 1.06, 95% CI 0.72–1.57) (34). Over 10 years of follow-up in the CREST trial, there was no significant difference in the rate of the primary end point between CAS group (11.8%) and CEA group (9.9%) (HR 1.10; 95% CI 0.83–1.44) (38).

Overall, there have been more periprocedural vascular events in CAS compared with CEA, but there was no significant difference in long-term results of randomized controlled trials. It is possible that the inclusion of asymptomatic patients could have offset the risk of high periprocedural events from symptomatic patients in the trials that enrolled both symptomatic and asymptomatic patients. In addition, we should consider the possibility that the difference in treatment effect was diminished by death from other causes in patients with higher atherosclerotic burden and comorbidity at the long-term follow-up. However, long-term prognosis and complications were not different in studies that followed patients for more than 2 years. Therefore, CAS seems to have the similar effect to CEA in the long-term period. We summarized the main randomized clinical trials comparing CEA and CAS in Tables 1 and 2. We did not include the CAVATAS study because only 25 patients (10%) actually underwent CAS among the endovascular group (*n* = 251) (31).

**Table 1:** **Major carotid revascularization trials comparing carotid endarterectomy with stenting**.

Trial	Sample size, *n* (CEA/CAS)	Old age, *n* (%)	Symptomatic (%)	Protection device (%)	30-Day total stroke (%)	30-Day composite outcome (%)	Follow-up period (years)	Long-term outcome; all stroke (%)	Long-term outcome; periprocedural stroke or death plus ipsilateral stroke postprocedural ipsilateral stroke or (%)
SAPPHIRE (32, 39)	167/167	19.5^a^	29	100	3.1/3.6	9.8/4.8^f^	3	10.7/10.1	10.2/9.0^i^
SPACE (28, 35)	584/599	21.6^b^	100	27	6.2/7.5	6.5/7.7^g^	2	10.1/10.9	8.8/9.5
EVA-3S (29, 37)	259/261	36.3^c^	100	92	2.7/8.7	3.9/9.6^g^	4	7.3/12.8	5.3/10.9
ICSS (13, 34)	858/855	53.3^d^	100	72	4.1/7.7^e^	5.2/8.5^e^	5	9.4/15.2	7.2/11.8
CREST (12, 33, 38)	1,240/1,262	9.6^a^	53	96	2.3/4.1	2.3/4.4^g^	4	7.9/10.2	4.7/6.4
CREST-S	653/668		100		3.2/5.5	3.2/6.0^g^	4	6.4/7.6	6.4/8.0
CREST-A	587/594		0		1.4/2.5	1.4/2.5^g^	4	2.7/4.5	2.7/4.5
ACT-1 (24)	364/1,089	0	0	100	1.4/2.8	2.6/3.3	1	2.2/3.3^h^	3.3/3.8^j^

*^a^The cutoff age for old age group is more than 80 years old*.

*^b^The cutoff age for old age group is more than 75 years old*.

*^c^The cutoff age for old age group is 75 or more years old*.

*^d^The cutoff age for old age group is 70 or more years old*.

*^e^A composite of stroke, death, or myocardial infarction within 120 days*.

*^f^A composite of death, stroke, or myocardial infarction within 30 days*.

*^g^A composite of death or stroke within 30 days*.

*^h^A composite of all stroke within 30 days plus ipsilateral stroke within 31 days to 1 year*.

*^i^A composite of stroke or death at 30 days plus ipsilateral stroke or death from neurological causes within 31 days to 3 years*.

*^j^A composite of death, stroke, or myocardial infarction within 30 days plus ipsilateral stroke within 31 days to 1 year*.

*SAPPHIRE, the Stenting and Angioplasty with Protection in Patients at High RIsk for Endarterectomy; SPACE, Stent-Supported Angioplasty of the Carotid Artery versus Endarterectomy; EVA-3S, Endarterectomy versus Angioplasty in Patients with Symptomatic Severe Carotid Stenosis; ICSS, International Carotid Stenting Study; CREST, the Carotid Revascularization Endarterectomy versus Stenting Trial; CREST-S, CREST symptomatic carotid stenosis group; CREST-A, CREST-asymptomatic carotid stenosis group; ACT-1, Asymptomatic Carotid Trial 1*.

**Table 2:** **Primary end point and definition of myocardial infarction in major carotid revascularization trials**.

Trial	Primary outcome	Definition of myocardial infarction for primary end point
SAPPHIRE (32)	A composite of death, stroke, or myocardial infarction within 30 days after the intervention or death or ipsilateral stroke between 31 days and 1 year	A CK level higher than two times the upper limit of normal with a positive MB fraction

SPACE (28)	Ipsilateral ischemic stroke or death from time of randomization to 30 days after the procedure	

EVA-3S (29)	The incidence of any stroke or death within 30 days after treatment	

ICSS (13)	The 3-year rate of fatal or disabling stroke in any territory	

CREST (12)	Stroke, myocardial infarction, or death from any cause during the periprocedural period or any ipsilateral stroke within 4 years after randomization	A CK-MB or troponin level that was twice the upper limit of the normal range or higher according to the left’s laboratory, in addition to either chest pain or symptoms consistent with ischemia or ECG evidence of ischemia, including new ST segment depression or elevation of more than 1 mm in two or more contiguous leads according to the core laboratory

ACT-1 (24)	The composite of death, stroke (ipsilateral or contralateral, major or minor), or myocardial infarction during the 30 days after the procedure or ipsilateral stroke within 1 year	Q-wave myocardial infarction—the development of new pathological Q waves in two or more contiguous leads with post-procedure CK or CK-MB levels elevated above normal
		Non Q-wave myocardial infarction—elevation of CK levels to greater than two times the upper limit of normal in the presence of elevated CK-MB and in the absence of new pathological Q waves. (WHO definition)

*SAPPHIRE, the Stenting and Angioplasty with Protection in Patients at High RIsk for Endarterectomy; SPACE, Stent-Supported Angioplasty of the Carotid Artery versus Endarterectomy; EVA-3S, Endarterectomy versus Angioplasty in Patients with Symptomatic Severe Carotid Stenosis; ICSS, International Carotid Stenting Study; CREST, the Carotid Revascularization Endarterectomy versus Stenting Trial; ACT-1, Asymptomatic Carotid Trial; CK, creatine kinase; ECG, electrocardiography; WHO, World Health Organization*.

In conclusion, CEA is recommended in patients with symptomatic carotid artery stenosis (>50%), especially in elderly patients when periprocedural complications are considered. CAS is indicated as an alternative to CEA for those who are asymptomatic, younger, and have higher operative risk. These trials have also shown that the efficacy of CAS was influenced by the patient’s age, medication, and the experience of the interventionists, and was similar to that of CEA in long-term follow-up.

## Age Issues for Selecting a Carotid Revascularization Method

The current guidelines, based on the results of previous clinical trials, emphasize that the presence of symptoms and the degree of stenosis are most important factors that impact the treatment of carotid stenosis (4, 5). Some review articles stated that patients aged 80 years or more are high risk for both CEA and CAS (Table 3) (40, 41). However, previous randomized clinical trials excluded patients over 80 years old, so the exact benefits and risks of CEA versus CAS in the very elderly are not well known. In subgroup analysis of NASCET and ECST, the risk of stroke and death in patients ≥75 years was higher in the medical treatment group than the CEA group and it suggested indirect favorable effects of CEA in the very elderly (42). In elderly patients, life expectancy is shorter than that of younger patients, so concerns about operative risk and vascular outcome are critical. However, patients in the medical arm had a higher risk of recurrent stroke, so the limitation of medical treatment used at the time of the trial also exists. Recent clinical trials comparing CEA with CAS included a substantial proportion of elderly patients. The rate of periprocedural and long-term vascular events was more prevalent in CAS group rather than CEA group, likely because of the vascular tortuosity and severe vascular calcification in the very elderly patients (Table 1) (43).

**Table 3:** **High risk condition or contraindication for carotid intervention of very elderly patients (40, 41)**.

	CEA	CAS
Anatomic	Prior CEA Prior neck surgery Prior neck irradiation Symptomatic ICA lesion High ICA lesion Low CCA lesion Neck immobility Tracheostomy Contralateral laryngeal nerve palsy Contralateral ICA occlusion Intraluminal thrombus Long subtotal ICA occlusion (string sign)	Symptomatic ICA lesion Steep aortic arch, tortuous CCA, tortous distal ICA Long subtotal ICA occlusion (string sign) Poor femoral access Extensive intracranial microvascular disease Circumferential ICA calcification Intraluminal thrombus Chronic ICA occlusion Intracranial aneurysm or AVM requiring treatment

Medical factors	Age > 80 Class III or IV heart failure Non-revascularized left main or multivessel coronary disease Class III or IV angina Myocardial infarction within 30 days Severe renal insufficiency Severe pulmonary disease Female sex Concomitant cardiac surgery Recent implantation of a coronary drug eluting stent	Age > 80 Severe renal insufficiency Major stroke within 4–6 weeks Intolerance to aspirin and/or clopidogrel

*CEA, carotid endarterectomy; CCA, common carotid artery; ICA, internal carotid artery; AVM, arteriovenous malformation*.

The age definition of “elderly” was different between studies. In meta-analysis, comparing early outcomes of CEA or CAS in old and young age groups was published in 2013 (44). Among 54 studies included, 38 studies used age 80 years as the cutoff for elderly, 13 studies used age 75 years, 2 studies used age 70 years, and another study used age 65 years. Of note, NASCET and CREST were the only RCTs included in the analysis, whereas the rest were observational studies. CAS was associated with increased incidence of stroke in elderly patients compared with younger patients [odds ratio (OR), 1.56; 95% CI 1.40–1.75], whereas CEA had equivalent cerebrovascular outcomes in old and young age groups (OR, 0.94; 95% CI 0.88–0.99). CAS had similar peri-interventional mortality risks in old and young patients (OR, 0.86; 95% CI 0.72–1.03), whereas CEA was associated with heightened mortality in elderly patients (OR, 1.62; 95% CI 1.47–1.77).

A pooled analysis of EVA-3S, SPACE, and ICSS showed that age was the only factor which significantly altered the relative risk of stroke or death between CAS and CEA in the short term (45). Whereas risk estimates were similar with both treatments among patients <70 years old, a twofold increase in risk with CAS over CEA was observed in the older age group. Age also significantly modified the effect of treatment on disabling stroke or death. The exploratory analysis of relative treatment risks across six age levels was consistent with the assumption of a linear increase in risk of periprocedural stroke risk associated with CAS. In CREST, there was an interaction between age and treatment efficacy (*p* = 0.02) (46). For the primary outcome, the hazard for CAS compared with CEA rose from 0.6 (95% CI 0.31–1.18) for patients aged under 65 to 1.08 (95% CI 0.65–1.78) for patients 65–74 years old to 1.63 (95% CI 0.99–2.69) for patients aged ≥75 years. The effect of age appeared primarily in the stroke risk, which increased with age more in the CAS group than in the CEA group. The age at which the HR was 1.0 was 70 years old for the primary outcomes and 65 years old for stroke.

In asymptomatic patients, the elderly (especially those over 80 years old) is a group in which the benefit of revascularization is controversial because in both the ACAS and ACST, the benefit from revascularization was seen after five-year follow-up (8, 20). ACAS did not enroll subjects greater than 80 years of age, and there was no benefit seen in those over 75 years in ACST. A report from the national cardiovascular data registry related to CEA showed elderly patients > 85 years of age were at increased risk for death or perioperative complications of stroke, death, and MI after CEA compared with those who were relatively younger. Among asymptomatic elderly patients, mortality rate was significantly higher in those older than 75 years (47). However, age cannot be an absolute contraindication with increasing life expectancy, because excellent outcomes after both CEA and CAS have been demonstrated in prudently selected patients (48, 49).

Therefore, if other conditions are similar, it is likely that CEA is more recommended than CAS for symptomatic patients older than 65–70 years old and it is difficult to determine which is better for asymptomatic patients in comparison with medical treatment. Meta-regression analysis investigating potential effects of publication time of each study on perioperative adverse events after CEA revealed no significant relationship. However, a significant effect of publication time on peri-interventional stroke and mortality in the patients who underwent CAS was reported (44). We need to consider comorbid medical conditions of the individual patients and wait for the results of research in progress.

## Risks and Specific Considerations for Carotid Revascularization of Very Elderly Patients

In elderly patients, it is necessary to know and confirm various situations that improve the outcomes after carotid revascularization. We reviewed specific considerations in very elderly patients undergoing CEA or CAS. However, we do not mention anatomic or medical conditions which is well known to be more appropriate for CAS or CEA in general population. We describe the conditions associated with an increased procedural risk and contraindications for CEA and CAS in Table 3 (40, 41).

### Sex

The effect of sex on carotid revascularization has been controversial. Previous studies such as NASCET and ECST showed a higher benefit in men than in women on perioperative stroke and death from CEA (42). Another study suggested that women were more likely to have less favorable outcomes, including surgical mortality, neurological morbidity, and recurrent carotid stenosis after CEA (50).

In general, surgical risk was higher in women than in men, whereas risk of CAS was virtually unaffected by sex (4, 51). There was no significant difference in treatment effects from CEA or CAS between men and women in the meta-analysis of randomized clinical trials of symptomatic patients (11). In CREST, the rates of the primary end point for CAS compared with CEA were similar, and there was no significant interaction between sexes (52, 53). However, periprocedural risk of events seems to be higher in women who have CAS than those who have CEA (6.8 versus 3.8%, *p* = 0.047) whereas there is little difference in men. A retrospective study based on a national database of carotid revascularizations in the United States found that women and men had equivalent rates of periprocedural stroke when undergoing CEA (1.0 versus 1.0%) and CAS (2.7 versus 2.0%) (54). Nevertheless, symptomatic women had a significantly higher rate of periprocedural stroke than symptomatic men (3.8 versus 2.3%; *p* = 0.03). Among those with symptomatic stenosis, there was no difference between CAS and CEA in periprocedural events among men, but there was a non-significant trend toward fewer events with women who received CEA versus CAS (3.4 versus 6.2%, *p* = 0.1). In asymptomatic women, rates of periprocedural strokes were significantly lower after CEA than after CAS (0.9 versus 2.1%, *p* < 0.001) Therefore, in elderly women, CEA is recommended rather than CAS.

However, the Italian study of carotid revascularization for patients greater than 80 years old showed a 5-year mortality of 49.4%, higher in males (39.5% for females and 52.5% for males) and ischemic stroke-related mortality of 20.2%, higher in females (40.0% for females and 15.6% for males) (55). Comparing data from octogenarian residents of the same geographical territory, ischemic stroke-related mortality hazard was significantly higher in the study females: OR 3.2 95% CI 1.16–9.17; *p* = 0.029 (for males: OR 0.97, 95% CI 0.89–1.10; *p* = 0.99). Five-year Kaplan–Meier estimates of any stroke was 84.8% (78.7% symptomatic versus 90.3% asymptomatic; *p* = 0.003). Therefore, invasive treatment of carotid stenosis may not be warranted in patients more than 80 years of age with carotid stenosis, especially when female and asymptomatic.

Because the proportion of women is higher in older patients, various medical conditions including age and sex should be considered together in the selection of CAS and CEA. In addition, for elderly women with asymptomatic carotid stenosis, some researchers asserted medical treatment is recommended as a priority (5, 23, 52).

### Coronary Artery Disease Risk

Because atherosclerosis is a systemic disease, coronary and carotid artery disease frequently coexist. The older the patient’s age, the greater the likelihood of coronary disease. The performance of either combined or staged coronary artery bypass grafting and CEA consistently has been associated with increased likelihoods of perioperative stroke, death, and MI compared with stand-alone CEA. In contrast, CAS among patients with concomitant severe coronary disease seems relatively safe in comparison with CEA (40).

Inclusion of MI in the primary end point was different across trials, and the results were influenced by this component (Tables 1 and 2). The SPACE and EVA-3S, which did not include MI for the composite outcome, failed to prove non-inferiority of periprocedural events in CAS group compared with CEA group (28, 29). However, SAPPHIRE, CREST, and ACT-1, which included MI in the primary end point, showed higher risk of MI with CEA during the periprocedural period (12, 24, 32). Especially in CREST, the rate of MI was higher in the CEA group rather than the CAS group (2.3 versus 1.1%, HR 0.50, 95% CI 0.26–0.94, *p* = 0.03) (12). In addition, both the patients with MI defined as biomarker elevation plus either chest pain or electrocardiography (ECG) evidence of ischemia and the patients with biomarker only (biomarker elevation with neither chest pain nor ECG abnormality) were independently associated with increased future mortality (56).

In some studies, the risk of MI did not increase with age in either treatment group (12, 44, 46, 47, 57). However, in other studies, postoperative MI after CEA or CAS was independently associated with older age (44, 58–60). A meta-analysis showed an increased risk of developing MI in older patients compared with younger patients after CEA [2.2 and 1.4%, respectively; OR 1.64 (95% CI 1.57–1.74)] and CAS [2.3 and 1.5%, respectively; OR 1.30 (95% CI 1.16–1.45)] (44).

To reduce the risk of MI after CEA or CAS, more detailed preoperative cardiovascular evaluation might be needed. Because asymptomatic coronary stenosis can influence periprocedural vascular events, intensive preoperative evaluation such as coronary computed tomography angiography can be helpful (61). In addition, patients in CREST undergoing CEA with regional anesthesia had a similar risk of periprocedural MI as those undergoing CAS, whereas the risk for CEA with general anesthesia was twice that compared with patients undergoing CAS (62). Thorough perioperative management and assignment to CAS instead of CEA may minimize ischemic cardiac complications even in elderly patients (63). In addition, the use of dual antiplatelet therapy, statins, cardioprotective pharmacotherapy with or without coronary revascularization, or regional anesthesia could be recommended in patients with coronary disease.

### Atrial Fibrillation

The prevalence of atrial fibrillation increases with age and ranges from 0.1% among adults less than 55 years of age to 9% in those ≥80 years of age (64). It is well known that atrial fibrillation increases the risk of stroke and is associated with poor stroke outcomes. In general practice, atrial fibrillation is a common comorbid condition among patients who undergo carotid revascularization. The rate of chronic atrial fibrillation in patients undergoing CEA ranges from 4 to 7% (65–67). Because many previous randomized controlled trials have excluded patients with cardiac arrhythmias (NASCET, ACAS, SAPPHIRE, CREST), the effect of atrial fibrillation on outcomes of patients is not well understood (6, 8, 12, 32).

Some studies revealed atrial fibrillation was associated with an increased risk of postoperative stroke in patients undergoing CEA but not in patients undergoing CAS. The relative risk of the composite outcome of postoperative stroke, cardiac complication, and mortality was increased in both groups with atrial fibrillation (65, 66). However, there were no long-term outcome results from the studies of CAS or CEA. The combined use of antiplatelets and anticoagulants in patients with atrial fibrillation after CAS may increase the bleeding risk, so additional studies are needed.

### Miscellaneous

The prevalence of concomitant severe steno-occlusion on the contralateral carotid artery and vertebrobasilar artery and/or aortic arch stenosis increases with age. Theoretically, patients with contralateral carotid or vertebrobasilar occlusion have an increased risk of intolerance to ipsilateral carotid clamping, distal cerebral embolization, or cerebral hyperperfusion syndrome. Aortic arch stenosis and severe angulation can induce technical failure of CAS. There have been conflicting data regarding the relationship between these concomitant atherosclerotic disease and risk of carotid revascularization (40, 68). However, recently both carotid revascularization methods can be applied in most patients, and final selection depends on individual situation.

White matter hyperintensities reflect small vessel burden and is associated with cognitive decline. The development of white matter lesions correlates with age. In a substudy of ICSS, CAS was associated with a higher risk of stroke compared with CEA in patients with moderate to severe white matter changes [an age-related white matter changes (ARWMC) score of 7 or more, HR for any stroke 2.98, 1.29–6.93; *p* = 0.011; HR for non-disabling stroke 6.34, 1.45–27.71; *p* = 0.014], but there was no difference in risk in patients with an ARWMC score of less than 7 (69). Therefore, it is necessary to be careful when selecting CAS in patients with more extensive white matter lesions. Nevertheless, there was a study reporting patients who underwent CAS tended to achieve higher scores in some cognitive function tests (70).

## Best Medical Treatment Versus Carotid Revascularization

Medical treatment with antihypertensive, single or multiple antiplatelets, and lipid-lowering drugs has advanced since most clinical trials have been completed comparing CEA with best medical therapy alone. Recent studies suggest that the annual rate of stroke in medically treated patients with an asymptomatic carotid artery stenosis has decreased (22, 23).

The population-based Oxford Vascular Study (OXVASC) assessed the risk of TIA and stroke in 1,153 patients with ≥50% carotid stenosis recruited consecutively in 2002–2009 (23). During 301 patient-years of follow-up (mean of 3 years), there were 6 ischemic events in the territory of an asymptomatic stenosis, 1 minor stroke, and 5 TIAs. The average annual rates on medical treatment were 0.34% for any ipsilateral ischemic stroke, 0% for disabling ipsilateral stroke, and 1.78% for ipsilateral TIA. A systematic review of the stroke risk in the medical treatment group of patients with severe asymptomatic carotid stenosis for the past 25 years was presented (22). The development of pharmacotherapy led to a decrease in the incidence of ipsilateral and total TIA or stroke since the mid-1980s. The risk of stroke in the medical arms in the post-2000 studies has been similar or lower than that of the CEA arms of major clinical trials in the 1990s.

Therefore, there is an urgent need for clinical trials comparing carotid revascularization therapy with optimal medical treatment in the patients with asymptomatic carotid stenosis. The aggressive medical treatment evaluation for asymptomatic carotid artery stenosis (AMTEC) study was the first randomized controlled trial comparing CEA and modern medical treatment (aggressive lipid-lowering and antihypertensive medication, and aspirin). However, the trial was stopped after a median follow-up of 3.3 years because of the obvious superiority of CEA (71). CREST 2 and SPACE-2 are now ongoing, and will compare intensive medical management alone versus CEA plus intensive medical management or CAS plus intensive medical management in patients who are asymptomatic (72–74). The results of these trials and a subgroup analysis of elderly patients may be helpful to answer the questions about best management of these patients.

Regardless, it is important to maintain optimal medical treatment in patients with carotid stenosis whether or not carotid revascularization procedures are performed. However, elderly patients may be at greater risk for side effects because adverse drug reactions and drug–drug interactions are more common in older patients than the general population due to polypharmacy, age-related changes in physiology, and/or underlying chronic disease (75). Aggressive antihypertensive treatment in elderly is highly associated with orthostatic hypotension, falls, and dementia (76). Hypoglycemia can lead to worse outcomes in elderly diabetic patients, which leads to a change in the recommended glycated hemoglobin levels (7.5–9% rather than <7%) (77). Therefore, prescription of these medications needs to be carefully performed in these elderly patients.

## Future Directions for the Patients with Carotid Stenosis—The Importance of Plaque

Carotid stenosis in the 60–99% range has been an inadequate predictor of future vascular events. The ACAS and ACST trials did not show stenosis degree in this range as a predictor of future events (8, 20). Therefore, stenosis degree alone is not a reliable predictor to be used in decision making, and carotid plaque could be an important factor to improve the prediction of future stroke risk.

The disruption of atherosclerotic plaques precedes the onset of a clinical stroke syndrome. Vulnerability of carotid plaques, characterized by cap rupture, is significantly associated with the development of vascular events such as stroke (78, 79). There were several studies that found clinical indices for identifying high risk carotid plaques. Multiple non-invasive imaging modalities have shown their potential to differentiate high-risk vulnerable plaques from stable plaques. Atherosclerotic plaques can be characterized based on their surface irregularity, ulcerations, echolucency and gray-scale values by ultrasound (80–82). The Asymptomatic Carotid Emboli Study (ACES) investigated 1 h transcranial Doppler recording from the ipsilateral middle cerebral artery, and the absolute annual risk of ipsilateral stroke or TIA between baseline and 2 years was 7.13% in patients with embolic signals and 3.04% in those without, and for ipsilateral stroke was 3.62% in patients with embolic signals and 0.70% in those without (83). MRI has also been used to detect the presence of intraplaque hemorrhage as indicative of a high-risk plaque and vessel wall imaging by high-resolution MRI can be helpful to differentiate vulnerable from stable plaques (84, 85).

Other studies using albumin-binding MRI and 18F-fluoride position emission tomography have also investigated detection of vulnerable plaques, and someday these efforts to differentiate stroke-prone patients will be helpful for the decision of carotid treatment (86, 87). In the future, these approaches will be used for personalized and precision medicine in the treatment of complicated elderly patients (88).

Better understanding of risk factors for periprocedural and long-term outcome would help clinicians offer the best treatment option for carotid artery stenosis in elderly patients. Because individual risks are different according to the treatment method and stroke incidence has been decreasing by advanced drug therapy and medical devices, clinicians should still be thoughtful while selecting patients for carotid revascularization therapy. Current guidelines may be changed in the future, depending on the results of ongoing trials of intensive medical management versus procedures plus medical management.

## Author Contributions

SH searched the literature, formulated the topics for the review, and wrote the manuscript. CB provided edits and contributed scientifically to the final draft.

## Conflict of Interest Statement

The authors declare that the research was conducted in the absence of any commercial or financial relationships that could be construed as a potential conflict of interest.
